# The preclinical pharmacology of the high affinity anti-IL-6R Nanobody® ALX-0061 supports its clinical development in rheumatoid arthritis

**DOI:** 10.1186/s13075-015-0651-0

**Published:** 2015-05-20

**Authors:** Maarten Van Roy, Cedric Ververken, Els Beirnaert, Sven Hoefman, Joost Kolkman, Michel Vierboom, Elia Breedveld, Bert ‘t Hart, Sofie Poelmans, Lieselot Bontinck, Alex Hemeryck, Sandy Jacobs, Judith Baumeister, Hans Ulrichts

**Affiliations:** Ablynx NV, Technologiepark 21, 9052 Zwijnaarde, Belgium; VIB, Rijvisschestraat 120, 9052 Zwijnaarde, Belgium; Crucell, Archimedesweg 4-6, 2333 CA Leiden, The Netherlands; Department of Immunobiology, Biomedical Primate Research Centre, Lange Kleiweg 161, 2288 GJ Rijswijk, The Netherlands; Department of Neuroscience, University of Groningen, University Medical Center, Hanzeplein 1, 9700 RB Groningen, The Netherlands

## Abstract

**Introduction:**

The pleiotropic cytokine interleukin-6 (IL-6) plays an important role in the pathogenesis of different diseases, including rheumatoid arthritis (RA). ALX-0061 is a bispecific Nanobody® with a high affinity and potency for IL-6 receptor (IL-6R), combined with an extended half-life by targeting human serum albumin. We describe here the relevant aspects of its *in vitro* and *in vivo* pharmacology.

**Methods:**

ALX-0061 is composed of an affinity-matured IL-6R-targeting domain fused to an albumin-binding domain representing a minimized two-domain structure. A panel of different *in vitro* assays was used to characterize the biological activities of ALX-0061. The pharmacological properties of ALX-0061 were examined in cynomolgus monkeys, using plasma levels of total soluble (s)IL-6R as pharmacodynamic marker. Therapeutic effect was evaluated in a human IL-6-induced acute phase response model in the same species, and in a collagen-induced arthritis (CIA) model in rhesus monkeys, using tocilizumab as positive control.

**Results:**

ALX-0061 was designed to confer the desired pharmacological properties. A 200-fold increase of target affinity was obtained through affinity maturation of the parental domain. The high affinity for sIL-6R (0.19 pM) translated to a concentration-dependent and complete neutralization of sIL-6R *in vitro*. In cynomolgus monkeys, ALX-0061 showed a dose-dependent and complete inhibition of hIL-6-induced inflammatory parameters, including plasma levels of C-reactive protein (CRP), fibrinogen and platelets. An apparent plasma half-life of 6.6 days was observed after a single intravenous administration of 10 mg/kg ALX-0061 in cynomolgus monkeys, similar to the estimated expected half-life of serum albumin. ALX-0061 and tocilizumab demonstrated a marked decrease in serum CRP levels in a non-human primate CIA model. Clinical effect was confirmed in animals with active drug exposure throughout the study duration.

**Conclusions:**

ALX-0061 represents a minimized bispecific biotherapeutic of 26 kDa, nearly six times smaller than monoclonal antibodies. High *in vitro* affinity and potency was demonstrated. Albumin binding as a half-life extension technology resulted in describable and expected pharmacokinetics. Strong IL-6R engagement was shown to translate to *in vivo* effect in non-human primates, demonstrated via biomarker deregulation as well as clinical effect. Presented results on preclinical pharmacological properties of ALX-0061 are supportive of clinical development in RA.

**Electronic supplementary material:**

The online version of this article (doi:10.1186/s13075-015-0651-0) contains supplementary material, which is available to authorized users.

## Introduction

Rheumatoid arthritis (RA) is a chronic, debilitating disorder with a prevalence believed to range from 0.5 to 1.0 % in the general population [1, 2]. Various disease-modifying antirheumatic drugs (DMARDs) have been in clinical use for decades to control the disease symptoms. However, there has been a paradigm shift in RA therapy during the past decades: current treatment aims at persistent and complete disease suppression, resulting in remission [1, 3, 4]. Although the use of tumor necrosis factor (TNF) inhibitors has revolutionized RA treatment in that aspect, a high number of patients still fail to achieve remission and do not show significant improvement [4]. Treatment response is thought to be heterogeneous in patients due to the relative dominance of a specific biological pathway or cellular phenotype [5, 6], and inhibition of the interleukin 6-interleukin 6 receptor (IL-6-IL-6R) axis has emerged as a powerful alternative, as demonstrated by tocilizumab (TCZ) [7, 8] and several other compounds in development [8].

IL-6 is a pleiotropic and key pro-inflammatory cytokine involved in the systemic inflammation and joint destruction observed in RA [9, 10]. The biological activity of IL-6 is mediated via a hexameric signaling complex, consisting of two molecules each of IL-6, IL-6R and glycoprotein 130. Formation of this complex leads to activation of the intracellular Janus kinase (JAK) / signal transducer and activator of transcription (STAT)-3, Ras/mitogen activated protein kinase (MAPK) or phosphoinositide 3-kinase (PI3K) / Akt pathway. Unlike other cytokines, IL-6 can initiate this signaling cascade through binding to either membrane-bound receptor (mIL-6R; classical signaling) or soluble receptor (sIL-6R; trans-signaling). IL-6 plays a critical role in different aspects of RA, such as the transition from the acute phase of inflammation to the chronic irreversible stage [11], stimulation of B cells to produce auto-antibodies, cartilage destruction [12] and anemia [13].

Nanobodies® are therapeutic proteins based on the smallest functional fragments of heavy chain-only (V_HH_) antibodies, naturally occurring in the Camelidae family [14–16]. In the present study we describe aspects of the preclinical development of the Nanobody® ALX-0061, consisting only of two domains which sufficed to confer the desired properties *in vitro* and *in vivo*. One domain of ALX-0061 was directed against IL-6R in order to inhibit the pro-inflammatory activities of the IL-6 pathway. The second domain was designed to improve the pharmacokinetic (PK) properties of the Nanobody by binding to human serum albumin (HSA). This format constitutes a minimized design, yielding a small structure which conveys high potency, as well as promising PK properties and *in vivo* efficacy. ALX-0061 was characterized using *in vitro* systems assessing affinity and potency. *In vivo* efficacy and pharmacodynamic (PD) properties were studied in an acute human IL-6 (hIL-6)-induced inflammation model in cynomolgus monkeys, and in a collagen-induced arthritis (CIA) model in rhesus monkeys.

## Methods

### Materials

ALX-0061 is a half-life extended bispecific Nanobody consisting of two sequence-optimized variable domains of llama-derived V_HH_ antibodies, directed against IL-6R and HSA, which were genetically fused via nine amino acids (GGGGSGGGS).

ALX-0061 and the monovalent anti-IL-6R domain were produced in a *Pichia pastoris* strain (Thermo Fisher Scientific, Waltham, MA) that expresses and secretes the Nanobody into the medium. The yeast cells were separated from the medium by centrifugation. The medium was subsequently clarified by depth filtration, after which the product was further purified using a process comprising three chromatographic steps. ALX-0061 was formulated in 15 mM L-Histidine (Sigma-Aldrich, St. Louis, MO), 8 % sucrose (234 mM; Fluka, Sigma-Aldrich, St. Louis, MO), and 0.01 % Tween-80 (w/w; Merck Chemicals, Darmstadt, Germany) and at pH 6.5. ALX-0061 was biotinylated (Pierce Biotechnology, Rockford, IL, USA), Alexa-fluor-647-tagged (Molecular Probes, Eugene, OR, USA), or sulfo-tagged (Meso Scale Discovery, Gaithersburg, MA, USA) according to the manufacturers’ instructions.

In the CIA study, clinical-grade TCZ (RoActemra®, 20 mg/mL; Roche, Basel, Switzerland) was administered to the rhesus monkeys undiluted at 0.5 mL/kg (10 mg/kg) as an intravenous bolus injection at indicated doses.

### Affinity maturation

The precursor of the anti-IL-6R domain of ALX-0061 was isolated from a llama immunized with recombinant hIL-6R (Peprotech, Rocky Hill, NJ, USA), and was subsequently humanized followed by affinity maturation.

In a first round of affinity maturation, three libraries of variants of the humanized Nanobody were screened. Diversity was introduced by substituting each residue of the complementarity determining region (CDR)1 and CDR2 with one or more residues with similar side-chain chemistry (library one), and in parallel by substituting with a panel of residues that naturally occur at the given position (library two). In a third library, each CDR3 residue was substituted with one or more residues with similar side-chain chemistries. Mutagenesis was performed by PCR overlap extension using degenerated oligonucleotides. The k_off_ values for target binding, as determined via surface plasmon resonance (SPR), were used to identify beneficial CDR mutations (see Kinetic analysis).

In a second round of affinity maturation, the beneficial mutations identified for all three CDRs were combined into a small library. Detailed k_off_ analysis of the second-generation Nanobody clones resulted in the selection of the anti-IL-6R domain of ALX-0061 for further characterization.

### Kinetic analysis

Kinetic analysis of ALX-0061/HSA interactions was performed on a Biacore 3000 instrument (Biacore International AB, Uppsala, Sweden). ALX-0061 was injected at concentrations between 10 and 100 nM onto CM5 sensor chips (Biacore, GE Healthcare, Little Chalfont, UK), containing immobilized recombinant HSA (Sigma-Aldrich, St Louis, MO, USA) after amine coupling. Dissociation was allowed for 300 seconds.

Kinetic analysis of the interactions of the different Nanobody variants with IL-6R was performed on a Biacore T100 instrument (Biacore International AB, Uppsala, Sweden). For k_off_ measurements, recombinant hsIL-6R was immobilized via amine coupling on CM5 sensor chips. Affinity maturation variants were injected onto the IL-6R surfaces at concentrations up to 200 nM. Dissociation was allowed for 30 minutes. For on-rate measurements, the non-neutralizing anti-hIL-6R monoclonal antibody (mAb) B-N12 (Diaclone, Besancon, France) was first immobilized on the sensor chip via amine coupling. Recombinant hIL-6R was captured at 100 nM, and the different Nanobody variants were injected onto the B-N12/IL-6R surfaces at concentrations between 6 and 200 nM.

Affinity analysis of ALX-0061/IL-6R interactions was performed with the Gyrolab™ Workstation using Gyrolab™ Bioaffy 1000 CDs (Gyros®, Upsalla, Sweden). The Gyrolab CD is a microscale (nanoliter) total analysis system with incorporated channels and structures, with immunoassay studies as the main present application. Capillary action and spinning is used for the desired incubation and washing steps. As a capture tool, 2000 nM of biotinylated recombinant hIL-6R was applied on the columns of the Bioaffy CDs, which were pre-packed with streptavidin-coated beads. A dilution series of hIL-6R was pre-incubated with a fixed concentration of ALX-0061 (50 pM) for 24 hours. This pre-incubation mixture was subsequently flown over the column, giving residual free ALX-0061 the opportunity to bind to hIL-6R immobilized on the column. Bound ALX-0061 was detected with 50 nM of an Alexa-fluor-647-labeled anti-Nanobody mAb. The fluorochromes were excited by a red laser, and the obtained fluorescent signals were amplified by a photo multiplier tube (PMT). Analysis was performed with the XL fit software of the Gyrolab workstation.

### Soluble receptor interleukin-6 neutralization potency ELISA

A non-neutralizing anti-IL-6R Nanobody (20 μg/mL) was immobilized on 96-well microtiter plates, after which the coated plates were blocked with Superblock T20 (ThermoFisher Scientific, Waltham, MA, USA). A dilution series of ALX-0061 in the absence or presence of 1 mg/mL HSA was first pre-incubated for one hour with 100 ng/mL recombinant hIL-6 (Gentaur, Kampenhout, Belgium) and 4 ng/mL recombinant hIL-6R, after which the mixtures were transferred to the coated wells. Bound IL-6R/IL-6 complexes were detected with 100 ng/mL of a biotinylated polyclonal anti-hIL-6 mAb (R&D Systems, Minneapolis, MN, USA), followed by horseradish peroxidase (HRP)-labeled streptavidin (Dako, Glostrup, Denmark, 1/5,000 or ThermoFisher Scientific, Waltham, MA, 1/25,000). Visualization was performed with esTMB (Stereospecific Detection Technologies reagents, Baesweiler, Germany), after which the coloring reaction was stopped with 1 M HCl. The absorbance was determined at 450 nm using 620 nm as reference wavelength.

### Plasma potency ELISA

The non-neutralizing anti-hIL-6R mAb B-N12 (5 μg/mL) was immobilized on 96-well microtiter plates, after which the coated plates were blocked with Superblock T20. A dilution series of ALX-0061, with 50 ng/mL recombinant hIL-6 and 50 % human plasma (Cryocheck^TM^, PrecisionBioLogic, Dartmouth, Canada) as a source of sIL-6R, was first pre-incubated for one hour and was then transferred to the coated wells. Residual hIL-6 bound to captured plasma hsIL-6R was detected with 0.2 μg/mL of a biotinylated polyclonal anti-hIL-6 antibody, followed by HRP-labeled streptavidin. Visualization was performed as described above.

### TF-1 cell-based proliferation assay

The TF-1 cell line (ECACC 93022307; Sigma-Aldrich, St. Louis, MO) was maintained between 0.5 and 9 × 100,000 cells/mL using RPMI 1640 medium supplemented with GlutaMAX (Invitrogen, Carlsbad, CA, USA), 10 % fetal bovine serum (Invitrogen), 1 % sodium pyruvate (Invitrogen), 1 % penicillin/streptomycin (Invitrogen) and 3 ng/mL human granulocyte-macrophage colony-stimulating factor (GM-CSF; eBiosciences, San Diego, CA, USA).

Cell suspensions were centrifuged, resuspended in culture medium without GM-CSF and seeded at a density of 12,500 cells/well in a 96-well plate. Cells were subsequently incubated for two hours with a dilution series of ALX-0061 in the absence or presence of 1 mg/mL recombinant HSA, after which 2 ng/mL recombinant hIL-6 was added and cells were further incubated for 72 hours at 37 °C in a humid chamber under a 5 % CO_2_ atmosphere. During the last six hours of the incubation, cells were pulse-labeled with 0.2 μCi/well of ^3^H-thymidine (GE Healthcare). Cells were then harvested and the ^3^H-thymidine incorporation was measured using a Topcount NXT counter (PerkinElmer, Waltham, MA, USA). Results were expressed as average counts per minute per well.

### Pharmacokinetic and pharmacodynamic study in healthy cynomolgus monkeys

This study was conducted in accordance with the requirements of UK law (Animals (Scientific Procedures) Act 1986) at the facilities of Covance Inc. (North Yorkshire, UK). The PK, immunogenicity (anti-drug antibodies (ADA)) and PD (free and total sIL-6R) of ALX-0061 were determined after a single intravenous (bolus or infusion) administration to male cynomolgus monkeys (*Macaca fascicularis*; Biodia Company Ltd, Port Louis, Mauritius). Dose levels were 1, 5, 10, 25 and 100 mg/kg and each group consisted of three animals. In addition, two animals were injected with formulation buffer and served as negative control. Blood samples were taken at pre-dose and up to 78 days post-administration.

Bioanalytical determination of total active ALX-0061 concentrations in plasma samples was performed using a Dissociation-Enhanced Lanthanide Fluorescent Immunoassay (DELFIA; developed at Ablynx, Gent, Belgium). In brief, a biotinylated bivalent anti-Nanobody Nanobody was captured onto streptavidin coated plates. Calibrators, quality control (QC) samples and study samples, complexed with hIL-6R, were detected with the europium-labelled non-neutralizing mAb B-N12. The fluorescent europium signal was measured on a Victor2V 1420 Multilabel Counter (PerkinElmer, Beaconsfield, UK).

Monkey plasma samples were further evaluated for the presence of anti-ALX-0061 antibodies using an electrochemiluminescent bridging assay (developed at Ablynx, Gent, Belgium). The same assay format was used for both screening and confirmation purposes. Briefly, biotinylated ALX-0061 was used to capture, and sulfo-tagged ALX-0061 was used to detect ADA in a homogenous assay format using a MSD Sector Imager 2400 (MesoScale Discovery, Rockville, MD, USA). Confirmation was performed by means of specificity testing, inhibiting the signal by competition with the drug. All samples were acid-treated before neutralization in the presence of biotinylated and sulfo-tagged ALX-0061.

Both total and free plasma sIL-6R concentrations were measured. Total plasma sIL-6R concentrations (that is, free sIL-6R and sIL-6R in complex with ALX-0061) were assessed using a sandwich ELISA (developed at Ablynx, Gent, Belgium). In brief, 96-well microtiter plates were coated with the capture non-neutralizing mAb B-N12, and excess binding sites were blocked with phosphate-buffered saline (PBS) plus 1 % casein. Calibrators, QC samples and study samples were applied on the plate, after 1/10 dilution in dilution buffer supplemented with 100 ng/mL ALX-0061 to overcome Nanobody interference. Bound sIL-6R was detected with a biotinylated polyclonal goat anti-hIL-6R antibody (R&D Systems), followed by HRP-labeled streptavidin. Visualization was performed as described above. Free plasma sIL-6R concentrations were measured using a Gyrolab™-based assay. In brief, the biotinylated monovalent anti-IL-6R domain of ALX-0061 was applied onto columns pre-packed with streptavidin-coated beads. Calibrators, QC samples and study samples were 1/10 diluted in Rexxip H buffer (Gyros) and applied onto the CD. After sample transfer, Alexa-fluor-647-labeled B-N12 was added at a concentration of 10 nM in Rexxip F buffer (Gyros). Analysis was performed as described above, optimized at 5 % PMT.

### Human interleukin-6-induced inflammation model in cynomolgus monkeys

The studies in this model were conducted using approved protocols, following applicable guidelines for the care and use of research animals (in accordance with European Council Directive of 24 November 1986 (86/609/EEG)) at the facilities of Laboratorium für Pharmakologie und Toxikologie (LPT; Hamburg, Germany).

A total of 14 adult cynomolgus monkeys were included in this study. ALX-0061 (0.4, 2 and 10 mg/kg) and its non-half-life extended monovalent anti-IL-6R domain (0.74 mg/kg) were administered on day 0 to three animals (two animals in the 10 mg/kg dose group) via a single intravenous bolus injection. Control animals (n = 3) received formulation buffer as placebo. Starting 24 hours after test item administration, all animals were injected subcutaneously once daily for seven consecutive days with recombinant hIL-6 (Gentaur) at 5 μg/kg.

Blood samples were taken pre-dose and up to 29 days post-administration for purpose of PK, ADA, PD (CRP and fibrinogen) and hematology (including platelet counts). Bioanalytical determination of total ALX-0061 plasma concentrations was performed using a sandwich ELISA. In brief, 96-well microtiter plates were coated with a bivalent capturing anti-Nanobody Nanobody (Ablynx, Gent, Belgium) and blocked with PBS plus 1 % casein (Thermo Fisher Scientific, Waltham, MA). After transfer of the calibrators, QC samples and study samples (1/10 dilutions in PBS plus 0.1 % casein) to the coated plate, the plates were incubated with a biotinylated detection anti-Nanobody Nanobody, followed by HRP-labelled streptavidin. Visualization was performed as described above.

Plasma concentrations of the monovalent anti-IL-6R binding domain were also measured using a sandwich ELISA. Neutravidin (Thermo Fisher Scientific, Waltham, MA) and a biotinylated anti-Nanobody Nanobody were used as capture, and Superblock T20 as blocking buffer. After transfer of the calibrators, QC samples and study samples (dilutions in PBS plus 0.1 % casein + hIL-6R for complexation with target) to the coated plate, detection was performed with the non-neutralizing mAb B-N12. The HRP-labelled polyclonal rabbit anti-mouse immunoglobulin (IgG) (DakoCytomation, Glostrup, Denmark) was used for visualization as described above. Data were expressed as ng/ml and individual plasma concentration-time profiles were subjected to a non-compartmental analysis (NCA) using the pre-programmed model 201 within WinNonlin version 5.1 (Pharsight, St Louis, MO, USA). The area under the curve (AUC) and derived PK parameters were calculated with the linear-up/log down trapezoidal rule.

### Collagen-induced arthritis study in rhesus monkeys

This study was conducted at the facilities of the Biomedical Primate Research Centre (BPRC; Rijswijk, the Netherlands) in accordance with the Dutch law on animal experimentation. The study protocol and experimental procedures were reviewed and approved by the Experimental Animal Care and Use Committee of the BPRC (approval number DEC #633) before the experiments started.

CIA-susceptible adult, healthy rhesus monkeys (*Macaca mulatta*; BPRC) were selected on basis of the absence of the dominant major histocompatibility complex (MHC) class I resistance marker *Mamu-B26*, as identified by serotyping [17–19]. CIA was evoked by immunization with 5 mg of chicken type II collagen (MD Biosciences, St Paul, MN, USA), dissolved in 0.1 M acetic acid to a final concentration of 10 mg/mL, and mixed with an equal volume of complete Freund’s adjuvant (CFA; DIFCO, Detroit, MI, USA).

A total of 19 animals were distributed over three groups. Prophylactic treatment was started before the onset of disease seven days after immunization. ALX-0061 (7.5 mg/kg; N = 7), TCZ (10 mg/kg; N = 7) or placebo (N = 5) was administered weekly, starting seven days after immunization. The dose for TCZ (10 mg/kg) was based on published data in a similar model in cynomolgus monkeys [20]. Test substances were given as an intravenous bolus injections. Formulation buffer of ALX-0061 was used as a placebo.

The animals were sedated twice weekly for assessment of clinical arthritis using the parameters of joint swelling, erythema, weight loss and impairment of movement. All clinical signs were graded on a scale from 0 to five, and resulted in an integrated discomfort clinical score using a semi-quantitative scale, as described previously [21–23]. The planned follow up period was 70 days after CIA induction, with animals being prematurely removed from the experiment for animal welfare reasons upon development of a pre-defined integrated discomfort clinical score [22, 23]. In addition to the clinical scoring, surrogate and biochemical disease parameters were determined twice weekly including body weight, swelling score and serum CRP levels, as described previously [21–23]. Blood was collected throughout the study for monitoring drug concentrations, and total sIL-6R was used as a biomarker indicating the presence of active drug, using ELISA-based assays as described above.

## Results

### Design of ALX-0061

ALX-0061 is a half-life extended bispecific Nanobody consisting of two sequence-optimized variable domains of llama-derived V_HH_ antibodies, genetically fused via nine linked amino acids (GGGGSGGGS). One domain provides the target specificity of ALX-0061, namely recognition of IL-6R, while the other interacts with HSA and mediates the half-life extension of ALX-0061. This constitutes a minimized design yielding a small two-domain structure with a molecular weight of 26 kDa. For comparison, a conventional therapeutic monoclonal antibody will comprise of 12 domains with a molecular weight of approximately 150 kDa [24].

The anti-IL-6R V_HH_ was obtained from a phage display library derived from a llama immunized with recombinant hIL-6R and following screening for sIL-6R binding and neutralization. Humanization of the V_HH_ was performed yielding a Nanobody domain with more than 90 % homology to the human germline consensus sequence in the framework regions. In order to further improve target affinity, this domain was submitted to two rounds of affinity maturation.

**Fig. 1:**
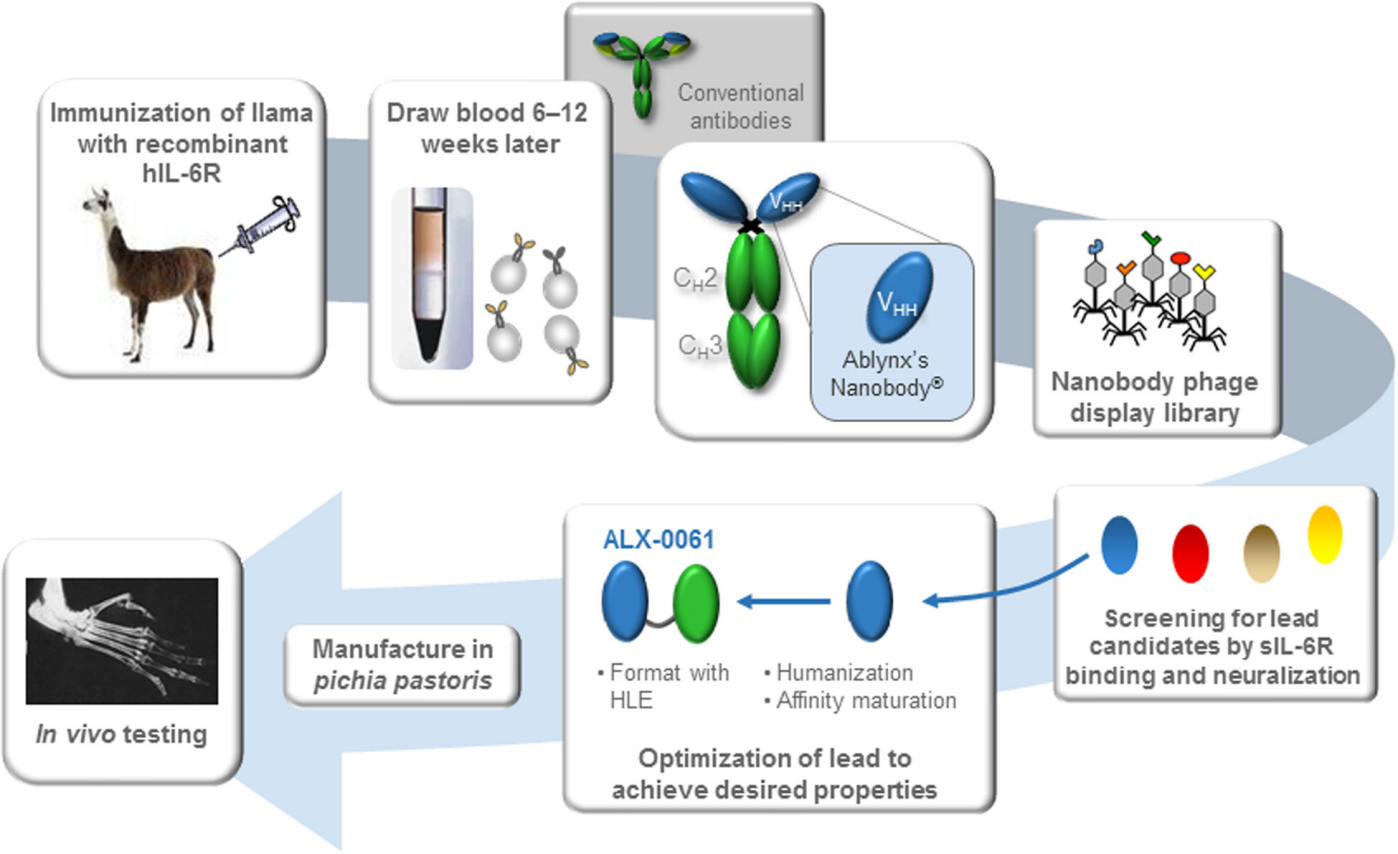
Development process of ALX-0061. After immunization of a llama with recombinant hIL-6R, a Nanobody phage display library was generated and screened for anti-IL-6R lead candidates. Optimization of the selected lead candidate consisted of humanization, yielding a Nanobody domain with more than 90 % homology to the human germline consensus sequence in the framework regions, and affinity maturation. Formatting of the selected lead anti-IL-6R domain to the HSA binding domain resulted in ALX-0061. After manufacturing in *Pichia pastoris*, ALX-0061 was tested *in vitro* and *in vivo*. hIL-6R: human interleukin-6 receptor; HLE: half-life extension; HSA: human serum albumin; sIL-6R: soluble IL-6R; VHH: heavy-chain-only antibodies

Kinetic parameters for binding of the parental and of five affinity matured variants to sIL-6R were determined via SPR. Due to the very slow dissociation of the affinity matured variants, only approximate values of the kinetic parameters could be derived. Affinity maturation resulted in an approximate 200-fold gain in affinity for sIL-6R compared to the parental Nanobody. Formatting of the selected lead anti-IL-6R domain to the HSA binding domain resulted in ALX-0061. The complete process from immunization to final format of ALX-0061 is presented in more detail in Fig. 1.

### *In vitro* characterization of ALX-0061

Due to the slow dissociation of ALX-0061 from hsIL-6R, the SPR methodology was not sufficiently sensitive for a reliable affinity determination. Therefore, the Gyrolab microfluidic-based analysis platform was used as an alternative platform to determine the equilibrium dissociation constant (*K*_*D*_) of ALX-0061 for IL-6R. The Gyrolab represents a novel platform alternative to determine compound affinity with, as its main advantage, the use of solution-based, and thus surface-free, measurements of unmodified molecules. An enhanced sensitivity can be obtained due to the increased surface-to-volume ratio. Using the Gyrolab Bioaffy microfluidic system, the *K*_*D*_ of ALX-0061 for hsIL-6R was determined to be 0.19 ± 0.08 pM (n = 7). Addition of saturating concentrations of 50 nM HSA had no significant effect on the affinity of ALX-0061 for hsIL-6R (data not shown). Affinity determination of ALX-0061 for HSA (mediated by its albumin-binding domain) was performed by SPR, yielding a *K*_*D*_ value of 22 nM.

**Fig. 2:**
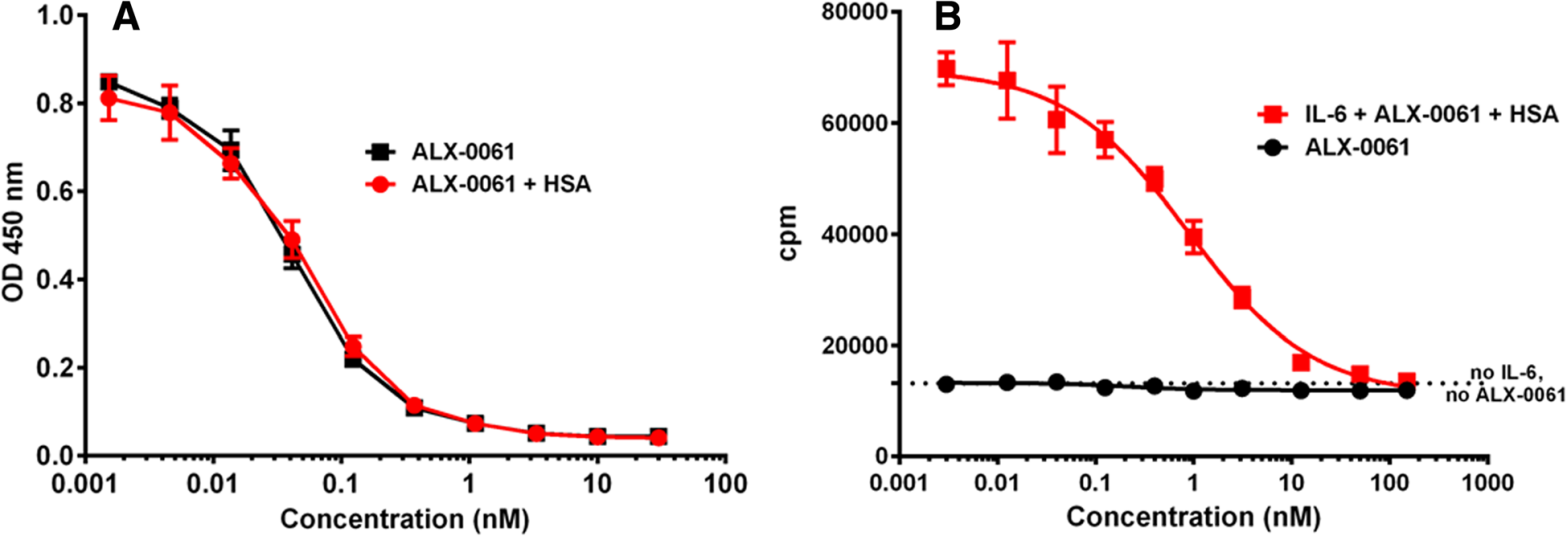
Inhibition profiles of ALX-0061 in an sIL-6R-based (**a**) and an mIL-6R-based (**b**) assay. ALX-0061 was pre-incubated with recombinant hIL-6 and recombinant hIL-6R, in the presence or absence of HSA, followed by the capture of hIL-6R on ELISA plates coated with a non-neutralizing anti-IL-6R mAb. IL-6 that remained in complex with IL-6R was detected using a biotinylated anti-IL-6 tool, and subsequent visualized with streptavidin-HRP. An example experiment for a neutralization experiment is presented in (**a**). Human TF-1 cells were pre-incubated with a dilution series of ALX-0061 in the presence of HSA, after which proliferation was induced with 2 ng/mL of IL-6. After 72 hours of incubation, cell proliferation was assessed by incorporation of ^3^H-thymidine. ALX-0061 did not induce proliferation in the absence of IL-6 (**b**). Symbols depict mean responses; error bars represent ± SD of triplicate samples within the experiment. ELISA: enzyme-linked immunesorbent assay; h: human; HRP: horseradish peroxidase; HSA: human serum albumin; IL-6: interleukin-6; IL-6R: IL-6 receptor; mAb: monoclonal antibody; mIL-6R: membrane IL-6R; OD: optical density; SD: standard deviation; sIL-6R: soluble IL-6R

The effect of ALX-0061 on target neutralization was further characterized *in vitro* using three functional assays that measure the interaction of IL-6 with its cognate receptor. In order to analyze possible effects of albumin binding on the IL-6R-blocking activity of ALX-0061, assays were also performed in the presence of saturating concentrations of HSA. In a first ELISA-based sIL-6R neutralization assay, ALX-0061, in a concentration-dependent manner, completely blocked the interaction of recombinant hIL-6 to recombinant hsIL-6R. The presence of HSA had no overt effect on the potency of ALX-0061 in this assay (Fig. 2a; Table 1). A similar observation was made in the plasma potency assay, which was used to evaluate the ability of ALX-0061 to prevent binding of recombinant hIL-6 to endogenous sIL-6R in the presence of endogenous HSA, in a pool of human plasma. Obtained potency values were comparable in both assays (Table 1). In a cell-based assay using human hematopoietic TF-1 cells, inhibition of IL-6-induced proliferation mediated by mIL-6R was analyzed. Also in this assay, ALX-0061, in a concentration-dependent manner, completely blocked proliferation of the TF-1 cells (Fig. 2b; Table 1). In contrast to what was observed in the ELISA-based sIL-6R neutralization assay, the presence of saturating concentrations of HSA had an effect, although minor (two to three fold), on the potency of ALX-0061 in this cell-based assay. In the absence of IL-6, TF-1 cell proliferation was not induced by ALX-0061 (Fig. 2b), confirming that ALX-0061 has no agonistic effect on TF-1 cells.

**Table 1:** *In vitro* characterization of ALX-0061 using functional neutralization assays

Assay	Target	IC_50_ (nM) without HSA	IC_50_ (nM) with 15 μM HSA
sIL-6R neutralization potency ELISA	recombinant sIL-6R	0.07 ± 0.02 (n = 5)	0.06 ± 0.01 (n = 3)
Plasma potency ELISA	endogenous sIL-6R	NA	0.26 ± 0.03 (n = 3)
TF-1 cell-based proliferation assay	mIL-6R	0.29 ± 0.03 (n = 3)	0.77 ± 0.18 (n = 3)

*In vitro* activity of ALX-0061 was assessed using three different assays. The mean IC_50_ ± SD is shown of multiple experiments, with the number of experiments indicated between brackets. 15 μM HSA should saturate ALX-0061 concentrations in these experiments. ELISA: enzyme-linked immunesorbent assay; HSA: human serum albumin; IC50: half minimal (50 %) inhibitory concentration; IL-6: interleukin-6; IL-6R: IL-6 receptor; mIL-6R: membrane IL-6R; NA: not applicable; SD: standard deviation; sIL-6R: soluble IL-6R

### Species cross-reactivity of ALX-0061

Both specificities of ALX-0061 (albumin binding and IL-6R blocking) were preserved in monkey species to enable nonclinical *in vivo* testing. Cross-reactivity was demonstrated for albumin of cynomolgus monkey, guinea pig, mouse and rat species (data not shown). Cross-reactivity of the IL-6R-targeting domain for membrane-bound and soluble forms of IL-6R from different species was evaluated *in vitro* by different approaches, including flow cytometry analysis, immunohistochemistry, and binding ELISAs. ALX-0061 was shown to be cross-reactive with cynomolgus and rhesus monkey species, but not with mouse, rat, and guinea pig sIL-6R. These data are summarized in an additional table (Additional file 1: Table S1).

### Effect of albumin binding on the pharmacokinetic behaviour of ALX-0061

**Fig. 3:**
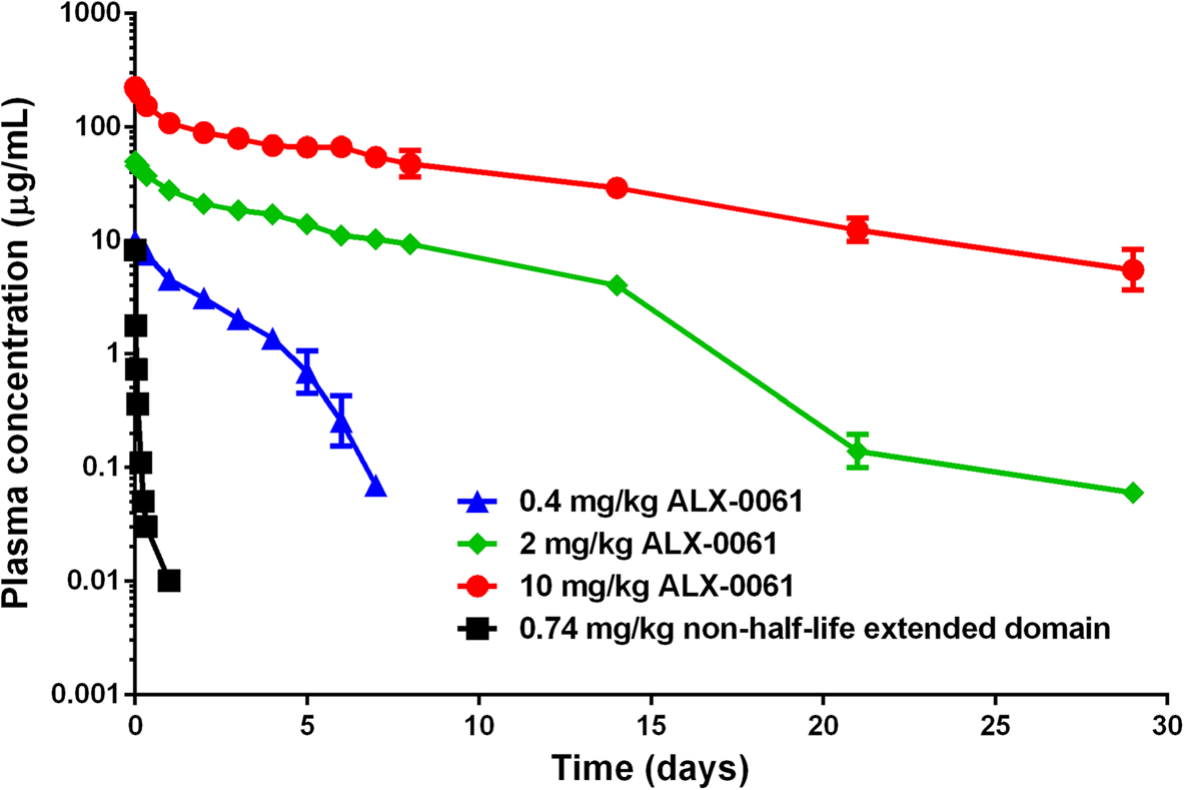
Plasma-concentration time profiles of anti-IL-6R Nanobodies after a single intravenous administration in cynomolgus monkeys. At day 0, animals received the monovalent non-half-life extended anti-IL-6R domain at 0.74 mg/kg (n = 3) or ALX-0061 at 0.4 (n = 3), two (n = 3), or 10 (n = 2) mg/kg, before daily subcutaneous injection with recombinant hIL-6 at 5 μg/kg for seven consecutive days. Mean (±SD) plasma-concentration time profiles of the Nanobodies are shown on a logarithmic scale. IL-6R: interleukin-6 receptor; SD: standard deviation

The influence of half-life extension via albumin binding was investigated in an IL-6-induced inflammation model in cynomolgus monkeys. The plasma-concentration time profiles after a single intravenous administration of 0.74 mg/kg of the monovalent anti-IL-6R domain or 0.4 mg/kg, 2 mg/kg, or 10 mg/kg ALX-0061 are shown in Fig. 3, and the corresponding PK parameters calculated by NCA are displayed in Table 2.

**Table 2:** Pharmacokinetic parameters of the non-half-life extended anti-IL-6R domain and ALX-0061 in cynomolgus monkeys

Test item	N	t_1/2, dominant_		t_1/2, terminal_		AUC_inf_		CL		V_ss_	
		(day)		(day)		(μg/day/ml)		(ml/day/kg)		(ml/kg)	
		Mean	CV %	Mean	CV %	Mean	CV %	Mean	CV %	Mean	CV %
Non-half-life extended domain i.v. 0.74 mg/kg	3	-	-	0.18	3	0.19	2	3971	2	227	27
ALX-0061 i.v. 0.4 mg/kg	3	1.73	11	0.53	6	16.3	14	24.8	14	42.8	8
ALX-0061 i.v. 2 mg/kg	3	5.00	15	1.44	9	193	5	10.4	6	53.7	7
ALX-0061 i.v. 10 mg/kg	2	6.61	11	-	-	1136	21	9.00	21	82.7	10

At day 0, three animals received the non-half-life extended domain at 0.74 mg/kg or ALX-0061 at 0.4, 2, or 10 mg/kg as a single intravenous dose, before daily subcutaneous injection with recombinant hIL-6 at 5 μg/kg for seven consecutive days. Pharmacokinetic parameters were calculated using non-compartmental analysis

i.v. = intravenous; t_1/2, dominant_ = dominant elimination half-life; t_1/2, terminal_ = terminal elimination half-life; AUC_inf_ = area under the concentration-time curve from time zero to infinity; CL = total body clearance; V_ss_ = volume of distribution at steady state. CV %: coefficient of variation; N: number of animals

The PK profile of the monovalent anti-IL-6R domain after a single intravenous administration showed a biphasic decline with a short apparent terminal half-life of 4.3 hours and a mean clearance of 3,971 mL/(day/kg). In contrast, the PK profiles of ALX-0061 showed a triphasic decline: a distribution phase was followed by a dominant elimination phase and a more rapid terminal elimination phase, and after increasing the dose from 0.4 to 10 mg/kg, the estimated clearance of ALX-0061 decreased (9.00 to 24.8 mL/(day/kg)) compared to its monovalent anti-IL-6R domain. The dominant elimination half-life of ALX-0061 increased along with the dosage (1.7 days after 0.4 mg/kg ALX-0061 to 6.6 days after 10 mg/kg ALX-0061), and the drug exposure increased more than dose-proportionally (Table 2, PK parameter AUC_inf_). The mean volume of distribution at a steady state ranged from between 43 and 83 mL/kg for the three ALX-0061 doses, suggesting that drug distribution is limited to the vascular and extracellular space, as generally described for monoclonal antibodies [25]. In summary, prolonged exposure was achieved with ALX-0061, whereas the non-half-life extended anti-IL-6R domain was rapidly cleared. These data provide evidence for the *in vivo* effect of half-life extension through binding to serum albumin.

### Pharmacodynamic activity of ALX-0061 in healthy cynomolgus monkeys

**Fig. 4:**
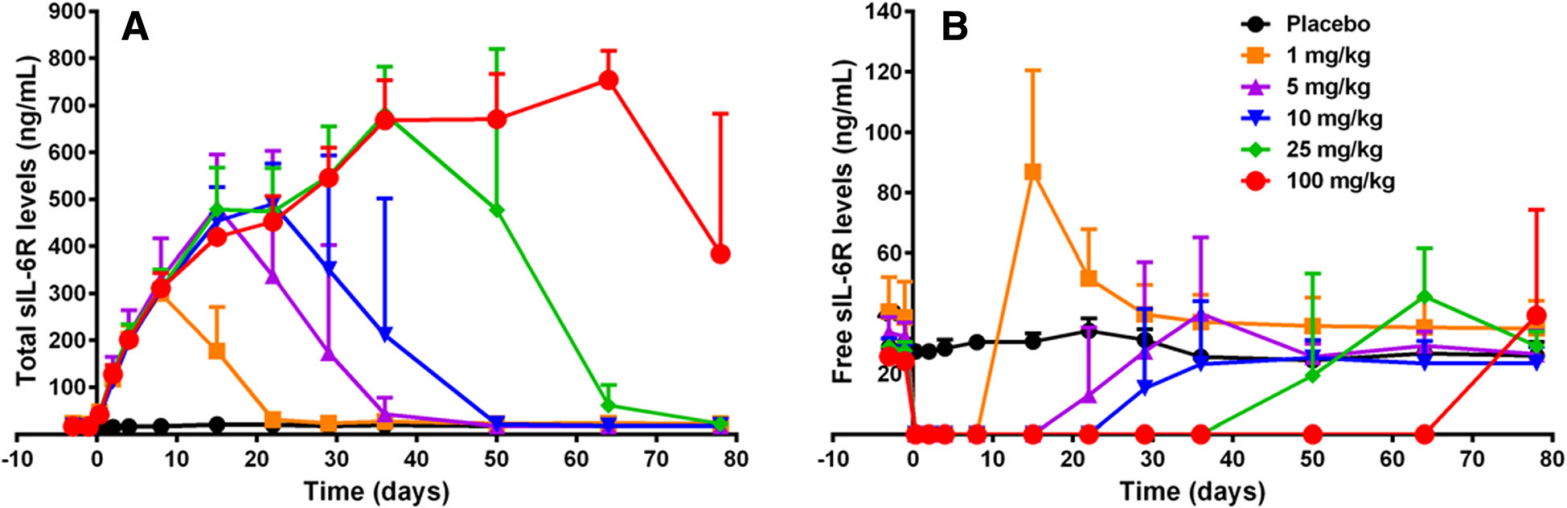
Effect of ALX-0061 on total (**a**) and free (**b**) sIL-6R plasma levels in cynomolgus monkeys. Per group, mean plasma concentrations of either biomarker ± SD are shown (n = 2 for vehicle group; n = 3 for dosing groups). Placebo or ALX-0061 at given doses were administered at day 0 as a single intravenous administration to non-stimulated cynomolgus monkeys. n: number of animals; SD: standard deviation; sIL-6R: soluble IL-6R

The prolonged exposure through albumin binding as described above translated into durable PD effects as was demonstrated in a PK/PD study. ALX-0061 showed a dose-dependent effect in cynomolgus monkeys (single intravenous doses ranged from 1 to 100 mg/kg) on: (i) the maximal total sIL-6R (that is, free sIL-6R and sIL-6R in complex with ALX-0061) concentrations during the observation period, (ii) the duration of baseline increased total sIL-6R concentrations, and (iii) the duration of the suppression of free sIL-6R (Fig. 4). In general, total and free sIL-6R concentrations returned to baseline levels after a given time, dependent on the dose. An association between an increase in total sIL-6R levels and a decrease in free sIL-6R levels was observed with longest elevation and suppression respectively in the highest dose group.

**Fig. 5:**
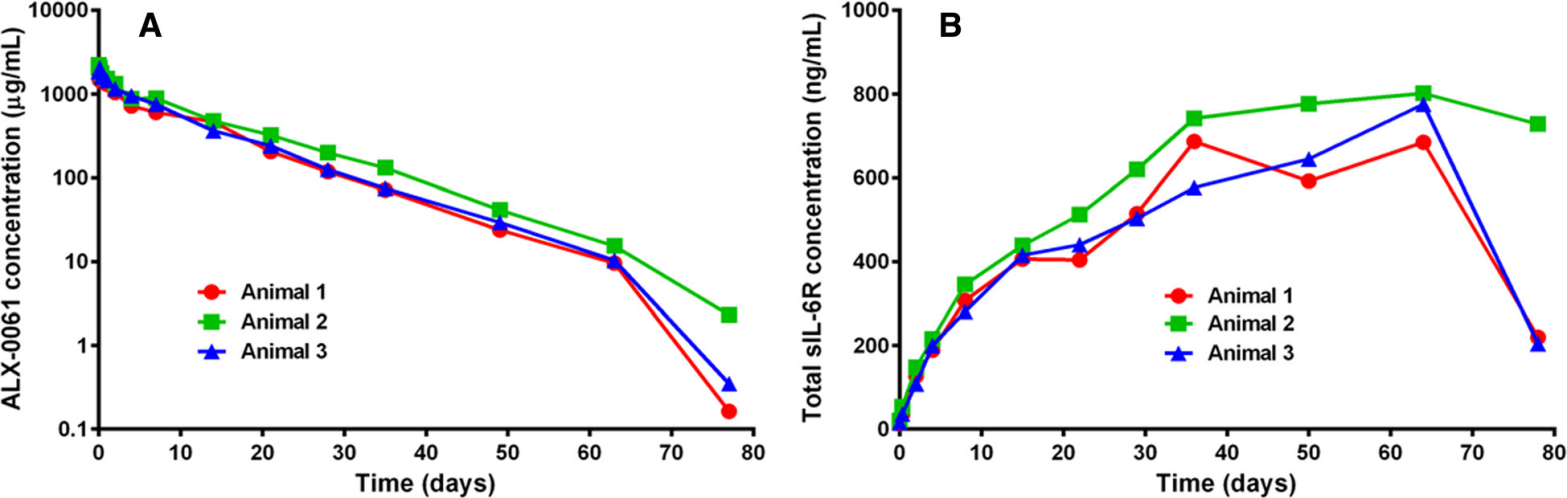
Total sIL-6R as PD marker for active drug exposure. Individual observed ALX-0061 (**a**) and total sIL-6R (**b**) concentration-time profiles are shown after a single intravenous administration of 100 mg/kg ALX-0061 in non-stimulated cynomolgus monkeys. PD: pharmacodynamic; sIL-6R: soluble IL-6R

The total sIL-6R concentration was shown to be a sensitive and timely marker for possible accelerated elimination of ALX-0061. This could be deduced from PK/PD data in animals showing aberrant PK profiles, most possibly due to the emergence of neutralizing and/or clearing ADA. Two animals in the highest dose group (100 mg/kg) showed a marked accelerated decrease in their ALX-0061 concentrations starting around day 63 (Fig. 5a), accompanied with a fast decline in total sIL-6R levels starting at the same time (Fig. 5b). Using a screening and confirmation assay, ADA was detectable in these animals within the same time frame, confirming possible neutralizing and/or clearing immunogenicity (data not shown).

### *In vivo* efficacy in a cynomolgus monkey model of human interleukin-6-induced inflammation

**Fig. 6:**
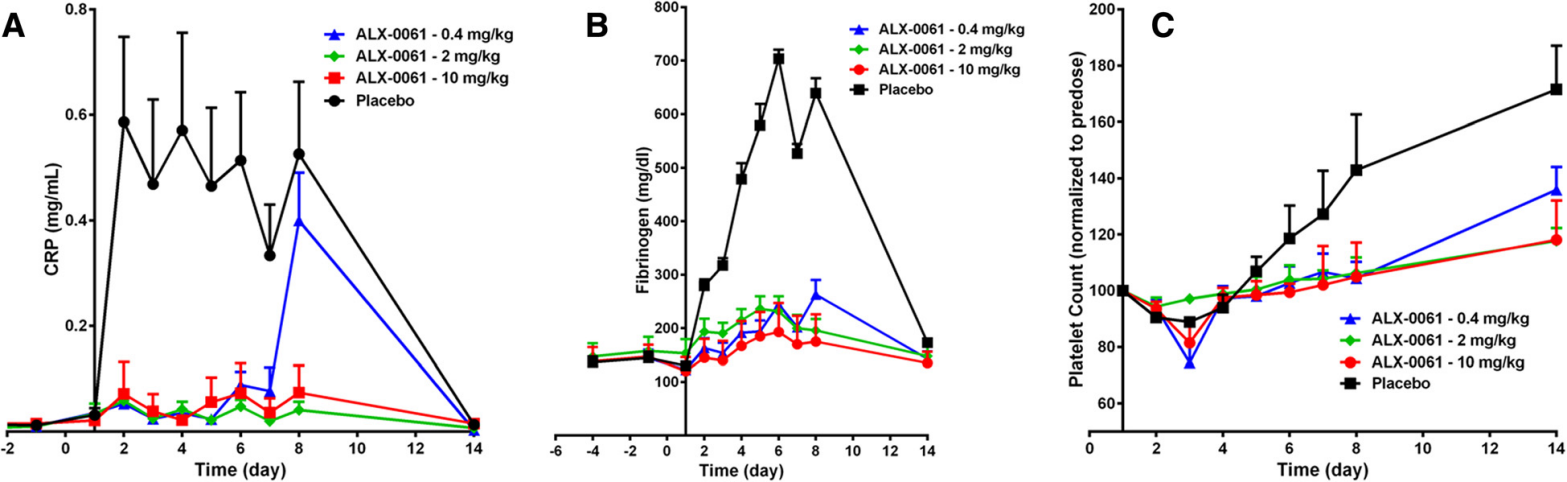
Acute phase response parameters in a cynomolgus monkey model of inflammation. Effect of a single dose of ALX-0061 (0.4, 2, or 10 mg/kg) on different acute phase response parameters is shown. (**a**) C-reactive protein plasma levels. (**b**) Fibrinogen plasma levels. (**c**) Platelet counts. Data were processed as follows: group mean values were calculated, error bars represent SEM, and platelets concentrations were normalized relative to individual baseline levels to reduce the inter-animal variation. CRP: C-reactive protein; SEM: standard error of mean

*In vivo* efficacy of ALX-0061 (administered at 0.4, 2, or 10 mg/kg) was first examined in a cynomolgus monkey model of hIL-6-induced inflammation. Plasma concentration of CRP, fibrinogen, and platelet numbers were monitored in the primates as inflammation parameters from the first hIL-6 injection until day 14. ALX-0061 conveyed *in vivo* biological activity, as indicated by a dose-dependent and complete inhibition of the three acute phase response parameters (Fig. 6a-c).

CRP concentrations increased rapidly after the start of hIL-6 injections in placebo-treated animals, and reached a maximum one day after the first administration (Fig. 6a). This effect plateau was sustained during the seven days of hIL-6 administrations. The hIL-6-induced increase in CRP was completely inhibited in the high (10 mg/kg) and middle (2 mg/kg) dose groups of ALX-0061. CRP induction was inhibited for seven days in the low (0.4 mg/kg) dose group, and levels were increased compared to baseline on day eight only. CRP concentrations returned to baseline levels once administration of hIL-6 was discontinued.

Fibrinogen concentrations increased five-fold compared to baseline in placebo-treated animals (Fig. 6b). Compared to the increase in CRP, the rise in fibrinogen levels exhibited a slower onset, and peaked five days after the first hIL-6 administration. In the high and middle ALX-0061 dose groups, fibrinogen induction by hIL-6 was completely inhibited. A minor increase in fibrinogen was observed in the low ALX-0061 dose group on day eight, which reached a maximum of two to three times the baseline level. Fibrinogen concentrations returned to baseline after discontinuation of hIL-6 treatment.

The number of platelets increased steadily in placebo-treated animals upon administration of hIL-6, and kept increasing after the last administration of hIL-6 at day seven, until the end of the experiment on day 14 (Fig. 6c). Some induction in platelet count was observed in the low dose group of ALX-0061, starting in all animals between day eight and 14, indicative of a suboptimal effect at this dose: maximal observed platelet counts in this group were around 120 to 150 % of baseline levels, compared to 140 to 190 % for placebo-treated animals. The effect of hIL-6 on platelet count was completely suppressed in the high and middle dose groups treated with ALX-0061. Lower and non-continuous exposure, such as those conferred by dosing the non-half-life extended variant, did not result in effects on the described biomarkers, as would be expected (data not shown).

### Collagen-induced arthritis model in rhesus monkeys

*In vivo* efficacy of ALX-0061, with respect to manifestations of clinical symptoms of RA, was further evaluated in a CIA model in rhesus monkeys, with inclusion of TCZ as a positive control. Acute or ‘early’ responders were first identified in the study based on the time interval between arthritis induction and a sharp increase in CRP or clinical score defined by: (i) an acute phase response of CRP (>50 mg/L) between day 10 to 14, or (ii) a clinical score of five before day 24 [22, 26]. Because of the violent and rapid onset of serious disease not reflective of a treatable clinical situation, early responders (four out of 19 animals) were excluded from certain analyses (Table 3).

**Table 3:** Overview of results obtained in the CIA study in rhesus monkeys

Compound	Animal ID	Day of CRP >50 mg/mL^a^	CRP maximum^b^ (mg/ml)	Day with Clinical score = 5^c^	Day of sacrifice^d^	Early responder	Neutralizing or clearing ADAs (based on PK and sIL-6R)	Body weight at day of sacrifice or day 70	Swelling score at day of sacrifice or day 70
Placebo	95020	24	547.5	NA	39	No	NA	-23 %	8
Placebo	R05029	21	191.5	NA	44	No	NA	-20 %	12.5
Placebo	R05053	24	535.6	NA	52	No	NA	-25 %	37
Placebo	R05058	17	547.2	NA	38	No	NA	-25 %	14
Placebo	R05073	14	643.1	28	29	Yes (CRP)	NA		
7.5 mg/kg ALX-0061	R00060	17	151.6	21	22	Yes (Cl Sc)	No		
7.5 mg/kg ALX-0061	R01016	35	101.9	35	34	No	Yes (Day 34)		
**7.5 mg/kg ALX-0061**	**R02055**	**NA**	**<50**	**NA**	**70**	**No**	**No**	**-10 %**	**0**
7.5 mg/kg ALX-0061	R03014	17	128.1	NA	49	No	Yes (Day 28)		
7.5 mg/kg ALX-0061	R05012	21	188.9	NA	44	No	Yes (Day 34)		
**7.5 mg/kg ALX-0061**	**R05042**	**NA**	**<50**	**NA**	**70**	**No**	**No**	**-15 %**	**2**
7.5 mg/kg ALX-0061	R06003	17	50.6	17	17	Yes (Cl Sc)	No		
**10 mg/kg tocilizumab**	**95031**	**NA**	**<50**	**NA**	**70**	**No**	**No**	**+4 %**	**0**
10 mg/kg tocilizumab	BB226	24	367.3	28	28	No	Yes (Day 28)		
10 mg/kg tocilizumab	R04042	28	448.1	31	31	No	Yes (Day 28)		
**10 mg/kg tocilizumab**	**R05059**	**NA**	**<50**	**NA**	**70**	**No**	**No**	**+6 %**	**0**
10 mg/kg tocilizumab	R05061	21	453.0	31	31	No	Yes (Day 24)		
10 mg/kg tocilizumab	R05089	28	161.7	NA	70	No	Yes (Day 28)		
10 mg/kg tocilizumab	R05090	21	171.1	21	21	Yes (Cl Sc)	Yes (Day 21)		

Treatment was started seven days after sensitization with chicken collagen type II. Animals were administered weekly for six consecutive weeks by intravenous bolus injection. ALX-0061 was given at 7.5 mg/kg, and TCZ at 10 mg/kg. Data were processed as follows: body weight was normalized to pre-dose weight (day 0); Body weight and swelling score are not included for early responders and animals which showed a clearing and/or neutralizing ADA response. Early responders were identified via CRP or clinical score. Animals which had active exposure throughout the study (that is, no neutralizing and/or clearing ADA) are highlighted in bold

^a^Study day that a CRP level of more than 50 mg/ml was measured for the first time, starting after day 10 (subsiding of the acute CFA response); increasing CRP levels after day 10 are interpretable as a sign of disease severity

^b^Maximum CRP values measurable for the given animal after day 10; these concentrations are interpretable as a measure of disease severity

^c^Study day on which a maximum clinical score of five was reported; this is interpretable as a measure of disease severity, with early onset being a hard-to-treat disease course

^d^Animals with high discomfort scores had to be euthanized during the study period

ADA: anti-drug antibodies; CFA: complete freund’s adjuvant; CIA: collagen-induced arthritis; Cl Sc: clinical score; CRP: C-reactive protein; ID: identification; NA: not applicable; PK: pharmacokinetics; sIL-6R: soluble interleukin-6 receptor; TCZ: tocilizumab

**Fig. 7:**
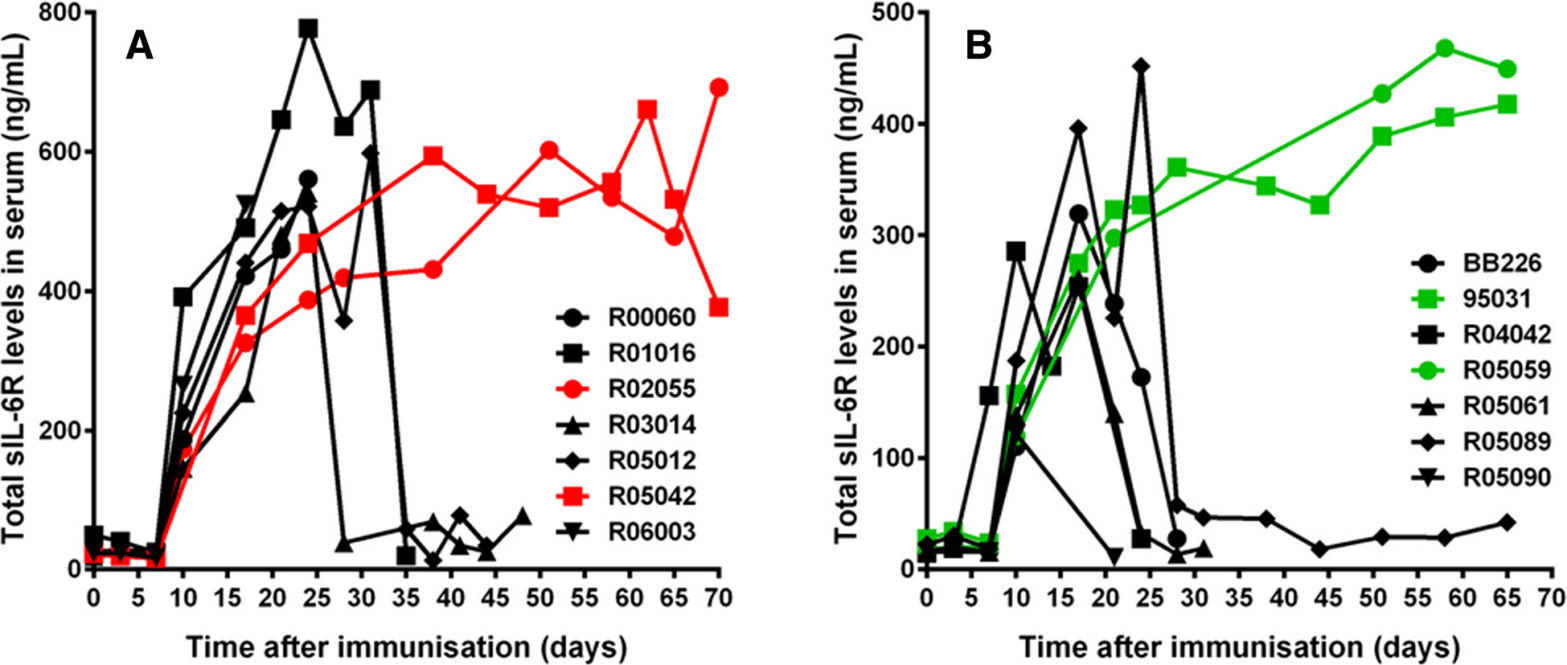
Total sIL-6R serum levels in the CIA study in rhesus monkeys. Curves depict results from individual animals. Acute responders and animals with neutralizing and/or clearing ADAs are indicated in black. Colored lines indicate animals with active drug levels throughout the entire study. Animals with high discomfort scores had to be euthanized during the study period. (**a**) Total sIL-6R levels after 7.5 mg/kg ALX-0061 treatment. (**b**) Total sIL-6R levels after 10 mg/kg TCZ treatment. ADA: anti-drug antibodies; CIA: collagen-induced arthritis; sIL-6R: soluble interleukin-6 receptor; TCZ: tocilizumab

It became apparent after PK/PD and immunogenicity assessment that interpretation of results was confounded by the appearance of immune responses that influenced the therapeutic effect of both TCZ and ALX-0061. Neutralizing or clearing ADA affected free active drug concentrations from day 20 onwards (that is, 13 days after the first drug administration). Both treatment groups were affected by ADA to the same extent (Table 3). Total sIL-6R levels increased rapidly after the first drug administration in all ALX-0061- or TCZ-treated animals, indicating the presence of active drug. However, total sIL-6R concentrations returned to baseline levels for five out of seven animals from either group, upon emergence of neutralizing and/or clearing ADAs (between day 24 and 34). Total sIL-6R levels remained elevated throughout the study in only two animals treated with ALX-0061 (R02055 and R05042) and two animals treated with TCZ (R05059 and 95031) (Fig. 7a,b), indicating exposure to active drug throughout the observation period in a minority of animals treated with ALX-0061 and TCZ.

**Fig. 8:**
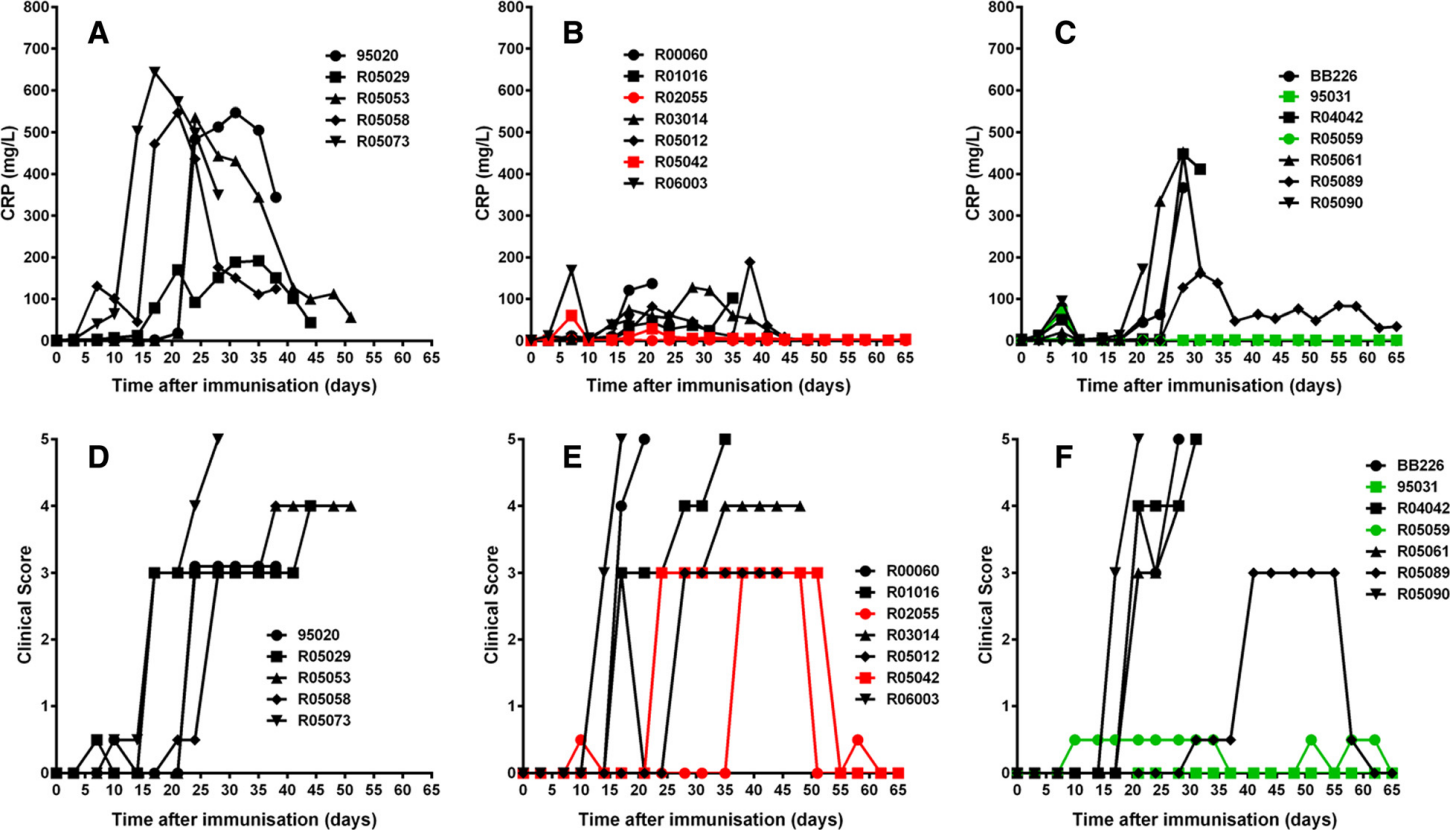
Effect of treatment on CRP and clinical outcome in the CIA study in rhesus monkeys. Curves depict results from individual animals. Acute responders and animals with neutralizing ADAs are indicated in black. Colored lines indicate animals with active drug levels throughout the entire study. Animals with high discomfort scores had to be euthanized during the study period. (**a-c**) Development of acute phase response as measured by CRP production. (**d-f**) Clinical score was calculated based on clinical signs, soft tissue swelling, body temperature, and body weight. Clinical score was monitored twice weekly. (**a, d**) Placebo; (**b, e**) 7.5 mg/kg ALX-0061; (**c, f**) 10 mg/kg TCZ ADA: anti-drug antibodies; CIA: collagen-induced arthritis; CRP: C-reactive protein; TCZ: tocilizumab

Several specific disease markers were measured in this study to obtain independent quantitative information on the systemic disease status, including CRP levels. Serum CRP levels are increased during the acute phase response of systemic inflammation, and directly reflect the intensity of the inflammatory process. IL-6R inhibition by ALX-0061 and TCZ resulted in a suppression of the CRP response, similar as was seen in the IL-6-induced inflammation study (Fig. 8a-c). Analysis of CRP plasma concentrations using normalized AUC values (AUC_norm_) to account for different follow-up periods demonstrated that, in the placebo group, significantly higher CRP concentrations were measured compared to treatment with TCZ (*P* = 0.015) or ALX-0061 (*P* = 0.011). No significant difference was noted between animals with or without interfering ADA, most likely because CRP is an early response marker, which is induced before the possible emergence of neutralizing ADA.

A pronounced reduction in the clinical severity scores, or a transient increase in the severity score with ensuing improvement of the condition, was observed in the animals which were exempt from an interfering immune response, and as a result, demonstrated an exposure to active drug throughout the observation period (Fig. 8d-f). Details of the obtained data are given in Table 3.

## Discussion

We present the novel biotherapeutic ALX-0061, consisting of two domains only, which we show suffices to confer favorable pharmacology *in vitro* and *in vivo*. ALX-0061 targets IL-6R and is currently in clinical development for the treatment of autoimmune diseases, such as RA [27]. Several compounds targeting the IL-6 pathway are currently in clinical development as a testament to the importance of the IL-6/IL-6R axis in chronic inflammatory diseases. This mode of action has been clinically validated by the development of TCZ, a monoclonal antibody against IL-6R, marketed as RoActemra® in Europe and Actemra® in the US and elsewhere. TCZ demonstrated good clinical responses and an acceptable safety profile in clinical trials, and continues to demonstrate this in daily clinical practice. The safety data are in some respects similar to those seen with other biologics in RA, including infections, although some IL-6 pathway-dependent effects are also seen, including a decrease in neutrophils and an increase in lipids, without a distinct cardiovascular safety risk. ALX-0061 is a bispecific Nanobody that has been designed for monovalent binding to hIL-6R, while its half-life in circulation is controlled by genetic fusion to a domain binding to HSA, one of the most abundant proteins in the blood. The results of this study demonstrate that this minimized structure of 26 kDa suffices to convey effective IL-6R neutralization *in vitro* and *in vivo*.

Compound-binding properties, such as affinity, and biological activity next to target selectivity contribute to the highly specific and competitive profile of therapeutic antibodies. ALX-0061 was purposely designed as a high-affinity binder to its receptor through affinity improvement *in vitro*, in particular to the soluble form of IL-6R. Typical Nanobody characteristics (such as small size, single-domain nature, and robust folding) make formatting and the construction of second-generation libraries based on a previously selected Nanobody more straightforward [14], which can be exploited to optimize binding characteristics to a given antigen more efficiently. The affinity of Nanobodies can be improved by generating bivalent Nanobody constructs that take advantage of avidity effects, without affecting the intrinsic affinity between a single Nanobody domain and its antigen, or by *in vitro* affinity maturation. In case of ALX-0061, approximately a 200-fold gain in affinity for sIL-6R compared to the parental Nanobody was achieved through affinity maturation. The *K*_*D*_ of ALX-0061 for IL-6R was quantified to be 0.19 ± 0.08 pM. To our knowledge, only a few studies describing a sub-picomolar affinity for therapeutic antibodies have been published [28, 29]. This research demonstrates that a single Nanobody target binding domain suffices and confers equal or higher affinity binding compared to published data of therapeutic monoclonal antibodies, where bivalent binding is conferred by four domains [24]. The high affinity of ALX-0061 translated in a high potency in ELISA-based neutralization assays for sIL-6R and in a cell-based neutralization assay for mIL-6R. Biologics with affinities in the femtomolar range may provide significant improvements in pharmacological activity, depending on the biological characteristics of the antigen, such as *in vivo* target and ligand concentration, as well as affinity for the ligand and disposition kinetics.

The affinity of ALX-0061 for HSA was determined via SPR, resulting in a *K*_*D*_ of 22 nM. This affinity for HSA can be expected to result in a high fraction of ALX-0061 bound to serum albumin *in vivo* considering the high abundance of albumin (approximately 640 μM in humans [30]). The albumin-targeting domain of ALX-0061 was confirmed to bind to cynomolgus monkey serum albumin, and thus was expected to provide persistent exposure: albumin is rescued from glomerular filtration and unspecific pinocytosis by binding to the neonatal Fc receptor (FcRn) in a similar way as antibodies, although an alternative binding site is used [31]. Results presented here confirmed that ALX-0061 shows PK properties in this species consistent with albumin-mediated FcRn recycling, while the non-half-life extended anti-IL-6R domain had only a short apparent terminal half-life of 4.3 hours. Presumably, the non-half-life extended domain is rapidly cleared via the kidneys, as its molecular weight of 13 kDa is expected to be well below the molecular weight cut-off for glomerular filtration, which is estimated at 70 kDa in humans [32, 33]. The mean clearance of the non-half-life extended domain was indeed in the same range as the glomerular filtration rate reported in monkeys (5 kg) of 2,995 mL/(day/kg) [34]. In contrast, the estimated clearance of ALX-0061 after single intravenous doses of 0.4 to 10 mg/kg was much lower, and can be attributed to binding of the HSA binding domain to serum albumin.

Furthermore, the estimated dominant half-life of ALX-0061 after 2 and 10 mg/kg intravenous administration (5.0 and 6.6 days, respectively) was similar to the estimated half-life of cynomolgus monkey serum albumin of 5.6 days, calculated for a 3 kg typical cynomolgus monkey, based on the relationship between albumin half-life and body weight (3.75/Body Weight(kg)^0.368^) described by Nguyen *et al*. [35]. A similar triphasic decline as was seen for ALX-0061 was also described for TCZ, whereby the rapid terminal elimination phase could be attributed mainly to target-mediated drug elimination due to IL-6R binding, which was saturated at high drug levels resulting in a lower overall clearance dominated by a linear non-saturable clearance (dominant elimination phase) [36]. Due to this non-linear PK behaviour of ALX-0061, estimated drug exposure and apparent half-lives were time- and dose-dependent.

The pharmacological activity of ALX-0061 towards IL-6R of the cross-reactive species cynomolgus and rhesus monkey was confirmed *in vivo* in two relevant non-human primate models. In an acute cynomolgus monkey model of hIL-6-induced inflammation, deregulation of IL-6R-dependent biomarkers was studied after prophylactic treatment and subsequent administration of recombinant hIL-6. ALX-0061 showed a dose-dependent and complete prevention of induction of three acute phase response parameters upon hIL-6 administration (CRP, fibrinogen, and platelet count), comparable to the results reported for TCZ in a similar model [37].

ALX-0061 was further evaluated in a rhesus monkey CIA model, in comparison to placebo and with TCZ as positive control. In this model, the induced disease closely resembles the pathological hallmarks of RA, including synovitis and joint destruction [23], and shows characteristic features of human RA as defined by the American Rheumatism Association criteria [23, 38]. *Mamu-B26* negative animals were selected before treatment start as rhesus monkeys with this genotype are highly susceptible for the inflammatory responses following collagen type II sensitization, and by pre-selection for this genotype, a CIA prevalence of >95 % can be achieved [17, 18, 23]. During the study, it became apparent from PK/PD and immunogenicity assessments that results in this particular model were complicated by the appearance of immune responses interfering with drug concentrations, and consequently therapeutic effect, from day 20 onwards (that is, 13 days after the first drug administration). All treatment groups were affected alike, and only two out of seven animals in the ALX-0061 group and two out of seven animals in the TCZ group remained available for full examination over the entire length of the study. Similar findings were reported in studies with other experimental therapeutic antibodies, such as daclizumab (anti-IL-2Rα) and PDL241 (anti-CD319). First, ADA were detected between 10 and 14 days in a prophylactic experiment [39], which was much faster compared to a study in naïve cynomolgus monkeys [40]. Moreover, the ADA titers were generally about five to 10 fold higher in the rhesus model compared to the naïve cynomolgus monkeys, and had a high impact on the plasma daclizumab levels [39].

In the PDL241 study, the development of a strong neutralizing ADA response was also observed in the majority of monkeys treated with the monoclonal antibody, with reduced exposure and strong infusion reactions as a consequence [19]. It is conceivable that in this rhesus model, the *Mamu-B26* negative genotype, possibly in combination with the complete Freund’s adjuvant in the inoculum, renders animals immunologically hypersensitive, as it does with chicken collagen type II. Although this immunogenic potential may depend on the class of drugs used and its mode of action, such as the anti-CD28 compound FR104 prevented the development of neutralizing antibodies [41], results obtained for biotherapeutics in the model need to be interpreted with care. An alternative CIA model in cynomolgus monkeys has been described and was used previously for TCZ [13, 20]. At the time, this model was not considered for the testing of ALX-0061 mainly due to animal welfare reasons; there seemed to be a gender bias and a higher number of monkeys is required as disease incidence is lower compared to the rhesus model [42, 43]. In addition, this model has also been described to be greatly influenced by immunogenicity: most of the animals showed an immune response to TCZ, and anti-TCZ antibodies had an impact on sIL-6R levels [13] or efficacy [20]. Other models, such as rodent models, could not be used due to lack of cross-reactivity.

In human RA, serum CRP levels are increased during the acute phase response of systemic inflammation, which precedes the onset of clinical symptoms and joint eroding processes, and they directly reflect the intensity of the inflammatory process [22, 23, 44]. Similar observations were made in the CIA model. Consequently, CRP concentrations were measured as a direct marker for early pathology. IL-6R inhibition by ALX-0061 or TCZ suppressed CRP induction in most of the animals as compared to the placebo group, and resulted in an early biological response before the emergence of the neutralizing ADA. As described above, the power of interpretation of clinically relevant parameters of arthritic disease was limited in this model. However, animals that were unimpeded by interfering ADA showed marked clinical treatment improvements. A similar subgroup analysis for efficacy was performed in the PDL241 study [19]. The effect of ALX-0061 on CRP seemed to be more pronounced than its clinical effect. Although both CRP and clinical effect are consequences of IL-6R inhibition, they have their own variability, with clinical effect being known to exhibit a higher variability compared to an acute marker as CRP. As a consequence, large studies would be necessary to draw statistical conclusions. CRP and other acute phase markers are also directly located in the IL-6 pathway as IL-6 is the main inducer of the acute phase response [45], which was confirmed by the results of the IL-6 induced inflammation model, while more parameters come into play to have an effect on clinical score. This was also seen with TCZ in clinical trials, in which 8 mg/kg TCZ demonstrated a fast and complete inhibition of CRP in most patients, although only approximately 30 % of these patients achieved clinical remission (DAS28 score <2.6) [46, 47].

The described non-human primate studies were used to confirm *in vivo* pharmacology as discussed above, but also to investigate possible biomarkers that may be translated to the clinical situation. These were in particular CRP, fibrinogen, and platelet counts as efficacy markers in a pathological condition, and total sIL-6R concentrations (that is, free sIL-6R and sIL-6R in complex with ALX-0061) as a PD marker both in healthy and diseased subjects. The robustness and suitability of sIL-6R levels as a proximal PD biomarker has been confirmed in the different nonclinical studies for ALX-0061 described here, and has been studied clinically for TCZ [48]. A transient increase in circulating sIL-6R could be observed upon administration of ALX-0061, reflecting an accumulation of sIL-6R/drug complex that assumes the long half-life of ALX-0061. The resulting sIL-6R increase and the duration of the effect showed a clear dose-dependent response. As ALX-0061 plasma concentrations decreased, concentrations of total sIL-6R decreased in parallel, as most of the total sIL-6R constitutes receptor in complex with drug. This was mirrored by suppression of free sIL-6R concentrations during treatment, which return to baseline upon elimination of ALX-0061 from the circulation. Full suppression of free sIL-6R concentrations demonstrated that measured total sIL-6R is indeed in complex with ALX-0061, and thus inactive. The good inverse association between total sIL-6R levels, free sIL-6R levels, and ALX-0061 concentrations confirmed that total sIL-6R is a valid and sensitive biomarker, indicating the presence of active drug, and a possible accelerated elimination of ALX-0061.

## Conclusions

ALX-0061 is a 26 kDa, bispecific, two-domain Nanobody targeting IL-6R and HSA simultaneously. This is the first report on the *in vitro* and *in vivo* activity of a Nanobody using this minimized design. ALX-0061 was purposely designed as a high-affinity binder for its therapeutic target through affinity maturation. Primary pharmacology, defined as neutralizing both forms of IL-6R (soluble and membrane-bound), was demonstrated *in vitro.* These observations showed that the high target affinity for IL-6R with a small biological entity translated into a high potency. Half-life extension of ALX-0061 was achieved by genetic fusion of the anti-IL-6R domain to an anti-HSA domain, and favorable PK/PD properties were confirmed *in vivo*. Possible biomarkers of effect were studied in different non-human primate models that allow correlation of PK, PD, and ADA. ALX-0061 is currently in clinical development, with promising results from a phase I/II trial in RA [27].

## Additional file

Additional file 1: Table S1. Species cross-reactivity of ALX-0061 towards sIL-6R and mIL-6R. Description of data: Species cross-reactivity was assessed qualitatively and quantitatively using different assays. ALX-0061 showed equal potency and binding towards IL-6R of non-human primates, and had a confirmed effect in nonclinical *in vivo* models. These data are summarized in Additional file 1: Table S1.

## Abbreviations

ADA: Anti-drug antibodies
CIA: Collagen-induced arthritis
CRP: C-reactive protein
ELISA: Enzyme-linked immunosorbent assay
h: Human
HRP: Horseradish peroxidase
HSA: Human serum albumin
IL-6: Interleukin-6
IL-6R: IL-6 receptor
*K*_*D*_: Equilibrium dissociation constant
mAb: Monoclonal antibody
mIL-6R: Membrane IL-6R
NCA: Non-compartmental analysis
PBS: Phosphate-buffered saline
PD: Pharmacodynamics
PK: Pharmacokinetics
QC: Quality control
RA: Rheumatoid arthritis
sIL-6R: Soluble IL-6R
SPR: Surface plasmon resonance
TCZ: Tocilizumab
V_HH_: Heavy-chain-only antibodies
