# Supplementary material for: The preclinical pharmacology of the high affinity anti-IL-6R Nanobody® ALX-0061 supports its clinical development in rheumatoid arthritis
Source: Arthritis Res Ther. 2015 May 20;17(1):135. doi: 10.1186/s13075-015-0651-0 (PMC4476083; doi:10.1186/s13075-015-0651-0)
Supplement: Additional file 1: Table S1. — Species cross-reactivity of ALX-0061 towards sIL-6R and mIL-6R. Description of data: Species cross-reactivity was assessed qualitatively and quantitatively using different assays. ALX-0061 showed equal potency and binding towards IL-6R of non-human primates, and had a confirmed effect in nonclinical in vivo models. These data are summarized in Additional file 1: Table S1. [file 13075_2015_651_MOESM1_ESM.docx]

**Additional file 1: Table S1: Species cross‑reactivity of ALX‑0061 towards sIL‑6R and mIL‑6R.** Species cross‑reactivity was assessed qualitatively and quantitatively using different assays. ALX‑0061 showed equal potency and binding towards IL‑6R of non‑human primates, and had a confirmed effect in nonclinical *in vivo* models.

| **Assay type** | **Test system** | **Human** | **Cynomolgus monkey** | **Rhesus monkey** | **Mouse** | **Rat** | **Guinea pig** |
| --- | --- | --- | --- | --- | --- | --- | --- |
| **Binding assays** | Flow Cytometry  (whole blood, mIL‑6R) | Mono+, granulo+, lympho+/‑ | Mono+, granulo+, lympho+/‑ | ND | No binding | ND | ND |
|  | Binding ELISA  (plasma sIL‑6R) | 0.13 nM ^a^  (n = 1) | 0.12 nM ^a^  (n = 1) | 0.27 nM ^b^  (n = 1) | No binding | No binding | No binding |
|  | Binding ELISA (sIL‑6R) ^d^ | 0.14 nM ^a^  (n = 1) | 0.25 nM ^a^  (n = 1) | ND | No binding | No binding | No binding |
|  | SPR (sIL‑6R) ^d^ | *K_D_* <14 pM | *K_D_* ~25 pM | ND | ND | No binding | No binding |
|  | Gyrolab (sIL‑6R) ^d^ | *K_D_* = 0.19  ± 0.08 pM  (n =7) | *K_D_* = 0.15  ± 0.05 pM  (n = 3) | ND | ND | ND | ND |
| **Functional assay** | Potency ELISA  (plasma sIL‑6R) ^c^ | 0.26  ± 0.03 nM  (n = 3) | 0.20 nM  ± 0.09 nM  (n = 2) | 0.30 nM  (n = 1) | No inhibition | No inhibition | No inhibition |
| ***In vivo* PD effect** | Total sIL‑6R levels (EC_50_) | NA | 2.6 nM | Biomarker and clinical effect | ND | ND | ND |
| **TCR** | IHC | Staining congruent with albumin and IL‑6R expression | Staining congruent with albumin and IL‑6R expression | ND | ND | ND | ND |

^a^IC_50_ value for competition with 0.4 nM ALX‑0061 (was confirmed with the monovalent anti-IL-6R domain)

^b^Measured for the monovalent anti-IL-6R domain

^c^IC_50_ value for neutralisation of IL‑6 binding to plasma sIL‑6R

^d^ recombinant material used

Abbreviations: TCR: Tissue-cross reactivity studies, IHC: Immunohistochemistry, NA: not available, ND: not determined, rec: recombinant, Mono: monocytes, Granulo: granulocytes, Lympho: lymphocytes; +: uniformly stained; +/‑: partly stained, SPR: surface plasmon resonance
